# cDCDD and Heart Procurement: Challenges from a French Critical Care Perspective

**DOI:** 10.3389/ti.2025.14779

**Published:** 2025-10-21

**Authors:** Matthieu Le Dorze, Julien Charpentier, Gaëlle Cheisson, David Couret, Guillaume Ducos, Benjamin Zuber

**Affiliations:** ^1^ Université Paris Cité, INSERM U942 MASCOT, Paris, France; ^2^ Department of Anesthesia and Critical Care Medicine, AP‐HP, Hôpital Lariboisière, Paris, France; ^3^ Service de médecine intensive‐réanimation, Hôpital Cochin, Assistance Publique – Hôpitaux de Paris, Paris, France; ^4^ Department of Anaesthesia and Intensive Care Medicine, Hôpital Bicêtre, AP‐HP, Le Kremlin‐Bicêtre, France; ^5^ Neurocritical Care Unit, University Hospital Saint Pierre, Réunion University, Saint Denis de La Réunion, France; ^6^ Reunion Island University, Institut National de La Santé Et de La Recherche Médicale, Diabète Athérothrombose Réunion Océan Indien, Saint Denis de La Réunion, France; ^7^ Department Bioethics, CERPOP, UMR 1295, Inserm - Université de Toulouse: “Trajectoires d’innovations en santé: enjeux bioéthiques et sociétaux”, Toulouse, France; ^8^ Medical Intensive Care Unit, Hôpital Foch, Suresnes, France

**Keywords:** controlled donation after circulatory death, end-of-life care, normothermic regional perfusion, dead donor rule, heart procurement

## Abstract

Controlled donation after the circulatory determination of death (cDCDD) is currently one of the most promising ways to increase organ availability. In France, a national cDCDD protocol requiring abdominal normothermic regional perfusion (A-NRP) has been in place since 2015. The recent consideration of heart procurement from cDCDD donors has reignited clinical and ethical debates within the critical care community. This position paper, endorsed by the two French intensive care societies, provides a critical care perspective on this evolving practice. Two key challenges are identified. First, heart procurement may require the withdrawal of life-sustaining measures (WLSM) to occur in or near the operating room, in contrast with French current practice where WLSM mostly takes place in the ICU. Intensivists strongly advocate maintaining ICU-based WLSM whenever possible, and ensuring continuity of care and end-of-life support when relocation is unavoidable. Second, the use of NRP raises concerns about the permanence of death and compliance with the dead donor rule. These concerns can be addressed through targeted biomedical research and a robust ethical framework affirming that death is declared prior to NRP and that no return to life is possible thereafter. Transparent engagement with these challenges is essential to sustain trust in the cDCDD pathway.

## Introduction

Heart procurement from donors after controlled donation following the circulatory determination of death (cDCDD) is currently under active consideration in France. Since the national implementation of cDCDD in 2015, a single standardized protocol mandating the use of abdominal normothermic regional perfusion (A-NRP) has been applied [1–3]. The potential extension of this program to include heart procurement–currently under review by the French regulatory authority (*Agence de la Biomédecine*) - opens a new chapter in the ongoing development of cDCDD in the country.

A central point of debate in the French context concerns the choice of surgical strategy for heart procurement in cDCDD donors. Two main approaches are currently under consideration: 1- direct heart procurement followed by *ex vivo* machine perfusion of the heart, in combination with A-NRP; and 2- thoraco-abdominal NRP (TA-NRP) with *in situ* restoration of cardiac activity prior to heart procurement [4–13]. Regardless of the strategy ultimately adopted, there is a strong national commitment to preserve the systematic use of A-NRP - given its proven benefits in terms of graft viability and post-transplant outcomes [14–19] - and to maintain a single standardized national protocol [2]. The choice between these two techniques requires careful consideration of multiple factors: their impact on recipients, including the viability and quality of both thoracic (heart and lungs) and abdominal grafts; the safety and feasibility of the procurement procedure itself; and the implications for donors, particularly regarding end-of-life care and compliance with the dead donor rule. In addition, broader technical, logistical, and financial aspects must also be carefully assessed when evaluating each approach.

However, this paper does aim to promote one surgical technique over another. At the time of writing, no definitive national decision has been made, and both strategies remain under review. Instead, this position paper - endorsed by the two French intensive care societies (*Société Française d’Anesthésie-Réanimation-SFAR, and Société de Réanimation de Langue Française-SRLF*) - aims to explore how the potential introduction of heart procurement in cDCDD has reopened two major issues already inherent to the cDCDD pathway: first, the potential impact of organ donation on end-of-life care; second, the debate surrounding the permanence of death and the compliance with dead donor rule when using NRP.

## Impact of Heart Procurement on End-of-Life Care

In the current French cDCDD protocol, the systematic use of A-NRP enables to withdraw life-sustaining measures (WLSM) within the intensive care unit (ICU), an environment familiar for both the patient and their relatives, prior to any post-mortem transfer to the operating room for organ procurement. Two exceptions to this practice have been observed: 1- in some centers, when lung retrieval is planned, WLSM may exceptionally occur in or near the operating room to meet the ischemic constraints of lung grafts; 2- when families explicitly expressed the wish not to be present at the WLSM time, some teams may opt for operating room to facilitate procedural logistics and optimize conditions for the installation of A-NRP and subsequent organ procurement. Nevertheless, more than 85% of WLSM in French cDCDD donors currently occur within the ICU. This is a major strength of the French protocol. It allows end-of-life care to be delivered in the patient’s usual care setting, ensuring relational continuity, geographical stability and the sustained involvement of the ICU caregivers that accompanied the patient and their family throughout their hospitalization. Within this model, organ donation minimally disrupts the dying process, therefore preserving the integrity of end-of-life care and supporting a patient-centered approach until death [20].

The introduction of heart procurement in French cDCDD donors will have a major impact on this current end-of-life care model. Regardless the technical approach used (direct procurement with A-NRP or TA-NRP), heart procurement requires that LSM be withdrawn in or near the operating room to meet the strict time constraints associated with the ischemic constraints of heart grafts (less than 30 min). Several challenges arise from this relocation. First, the physical environment of the operating room is inherently technical and not designed to support the emotional needs for the patient’s families during the dying process. The presence and involvement of relatives becomes difficult, if not impossible. Second, the ability of ICU caregivers–particularly nurses–to accompany the patient is considerably reduced. Finally, this relocation risks reinforcing a technical and time-driven approach to dying, in which organ donation take precedence over a patient centered end-of-life care.

The potential introduction of heart procurement in French cDCDD donors has reaffirmed the position of intensivists regarding the appropriate location for WLSM. From the perspective of ICU caregivers, WLSM should, whenever possible, take place in the ICU. The only acceptable reason for relocating this step in or near the operating room is the need to meet ischemic constraints specific to certain grafts, particularly the heart and lung. Conversely, the absence of relatives at the time of WLSM should not, in our view, justify such relocation. Even in their absence, the ICU provides a more supportive environment for dying, ensuring continuity of care and the presence of familiar caregivers, particularly nursing staff.

Although relocation to the operating room may occasionally be necessary, the associated challenges can be addressed through targeted organizational and training strategies. Dedicated spaces adjacent to the operating room should be created to enable the presence of relatives and ICU caregivers. Furthermore, all healthcare professionals involved, including anesthesia and surgical teams, should receive trainings to maintain the quality of end-of-life care, facilitate the presence of relatives when appropriate, and foster effective collaboration with ICU teams.

## Impact of Heart Procurement in cDCDD Donors on the Permanence of Death and on the Dead Donor Rule

The permanence of death and compliance to the dead donor rule when using NRP represent central clinical and ethical challenges in the development of cDCDD. These issues have been widely explored in the international literature [4, 21–25]. The following section summarizes the main dimensions of this debate before exploring how these questions are addressed from a French critical care perspective.

There is currently a broad international scientific and medical consensus that defines death as the permanent loss of brain function, that is the complete absence of consciousness brainstem reflexes, including the ability to breathe spontaneously [26, 27]. This definition has its origin in the concept of brain death, developed in the 1960s, which enabled both the possibility of withdrawing LSM in ICU patients in an “irreversible coma,” and the possibility of organ donation from donors with a beating heart [28–31]. Importantly, this definition is based on the notion of *permanence,* rather than *irreversibility*. Brain function is considered permanently lost if it will not return spontaneously and will not be restored by intervention. In contrast, irreversibility implies that brain function cannot be restored even if an intervention were performed [32, 33]. In addition, this definition emphasizes the loss of brain *function* rather than the cessation of cerebral *circulation* [30, 31]. Two pathways of dying are considered. In the circulatory sequence, the permanent cessation of peripheral circulation leads to the permanent cessation of cerebral circulation, which the results to the permanent loss of brain function. In the neurological sequence, a devastating brain injury leads to the permanent cessation of cerebral circulation due to intracranial hypertension, resulting in the loss of brain function [27]. The dead donor rule is a fundamental ethical principle in organ donation. It is based on two core requirements: first, that organs may only be retrieved from patients who have been declared dead using accepted medical criteria; and second, that organ procurement must not cause the patient’s death [34–38].

However, this physiological and ethical framework is challenged by the use of NRP in cDCDD, both in A-NRP, which is currently used in France, and in TA-NRP, a technique that may be selected for introducing heart procurement into the French cDCDD protocol. In the French protocol, as in other international cDCDD protocols, death is declared after a clearly defined sequence [2]. Following the WLSM, circulatory arrest is confirmed by the absence of arterial pulsatility. This leads to the cessation of cerebral circulation and the complete loss of brain function. After a five minutes no-touch period, the loss of brain function is considered permanent, and death is declared [39, 40]. In the current French cDCDD protocol, A-NRP is then initiated to restore circulation to abdominal organs to improve graft viability and function. To prevent any restoration of cerebral circulation, an intra-aortic balloon is used to maintain the permanence of brain function loss and, therefore, the validity of death determination.

While this sequence is clearly described, its integrity can be challenged. When using A-NRP or TA-NRP, several technical and anatomical factors may compromise the exclusion of cerebral circulation. Intra-aortic balloons may be insufficiently occlusive, allowing the restoration of coronary circulation, which may in turn lead to the resumption of cardiac activity, followed by the restoration of peripheral and cerebral circulation, and ultimately the restoration of brain function–a function that was deemed permanently, but not yet irreversibly, lost. In some cases, cerebral circulation may be restored more directly, even in the absence of cardiac activity restoration. Furthermore, collateral circulation between the thoracoabdominal aorta and the posterior cerebral circulation, as well as anatomical variants, may allow blood to bypass the occlusion created by balloons or vascular clamps. This can lead at least a partial restoration of posterior cerebral circulation, and therefore of brain function, particularly the brainstem function [4]. Such scenarios directly challenge the first requirement of the dead donor rule–namely that organs can only be retrieved from patients who have been declared dead. Moreover, the use of intra-aortic balloon or vascular clamps raises ethical concerns regarding the second requirement of the dead donor rule - namely that organ donation process must not cause death. By actively preventing the potential restoration of cerebral circulation, the technique may be perceived as ensuring that death occurs, rather than simply confirming that it has taken place [41].

The perspective of introducing heart procurement from cDCDD donors has recently triggered renewed debate in France regarding the permanence of death when using NRP. Interestingly, this issue has attracted little attention at the time of the initial implementation of the French national cDCDD protocol in 2015, despite the protocol mandating the systematic use of A-NRP. At that time, the French critical care community was primarily focused on other critical aspects of the protocol, particularly the potential impact of organ donation on end-of-life decision-making [42, 43]. In this new phase, however, the potential introduction of TA-NRP as part of heart procurement protocol has brought to highlighted medical and ethical concerns regarding the permanence of death when using NRP in cDCDD donors. In response, French intensivist, supported by the two French intensive care societies, advocate for a combined approach based on both biomedical evidence and ethical deliberation. This has involved both a critical review of the medical literature in accordance with evidence-based medicine, and the facilitation of structured spaced for interdisciplinary discussion involving ethicists.

From a biomedical perspective, several research strategies are currently being explored to provide evidence of the permanence of brain function loss when using NRP [24]. The first strategy seeks to identify technical solutions that would completely prevent any restoration of the cerebral circulation. However, this remains limited, as collateral circulations and anatomical variants, may still permit some degree of cerebral circulation. The second strategy aims to determine the point at which brain function loss becomes irreversible, either by establishing a time threshold beyond which recovery is impossible, or through the development of neurological monitoring tools. The third strategy focuses on identifying the minimal level of cerebral blood flow, in terms of flow or perfusion pressure, below which the permanent (though not yet irreversible) loss of brain function cannot be restored. This physiological threshold remains poorly understood and is likely patient-specific. The fourth strategy seeks to demonstrate the permanence of the complete loss of brain function during NRP despite a possible partial restoration of cerebral circulation, particularly posterior circulation. This approach, however, is limited by current monitoring tools and by the systematic use in France of continuous and deep sedation maintained until death, a confounding factor in the assessment of brain function [44, 45].

Although complex, this biomedical agenda is seen as necessary, and research is ongoing. Available data are rather reassuring [4, 46–50]. One particularly informative study directly monitored pressures at different anatomical sites during NRP, including the radial artery (reflecting thoracic pressure), the femoral artery (abdominal pressure), and the intracranial arterial pressure at the circle of Willis. In two TA-NRP procedures performed with median sternotomy, ligation of the three arch vessels, and venting of the cephalad ends to the atmosphere, no measurable intracranial pressure was detected when NRP was initiated, despite restoration of thoracic circulation and return of cardiac activity [48]. These results support the hypothesis that appropriate surgical techniques can effectively prevent cerebral reperfusion during NRP, thereby helping to address ethical concerns related to the dead donor rule and supporting the expansion of cDCDD programs. Nevertheless, important gaps in our physiological knowledge remains. First, the temporal sequence linking circulatory arrest, the permanent loss of brain function, and its irreversibility is still not fully understood. Second, the precise thresholds - whether in terms of cerebral blood flow or perfusion pressure - below which brain function becomes permanently and irreversibly lost have yet to be clearly defined and are likely to vary between individuals. Far from being a limitation, these challenges represent a major opportunity to strengthen the scientific foundations of cDCDD and improve the safety and acceptability of its protocols. Continued interdisciplinary research is therefore both necessary and promising.

The issue related to the permanence of death when using NRP must also be assessed from an ethical perspective [21, 22, 51–54]. While some have argued that NRP *de facto* violates the dead donor rule, the ethical approach, in our view, must follow a completely different path, based on two key considerations. First, the decision to withdraw LSM has been made solely in the best interests of the patient, independently of any consideration for organ donation. The dying process is therefore initiated for clinical and ethical reasons unrelated to transplantation. Second, the potential cDCDD donor is, in our view, indeed dead at the time of organ procurement, despite the limitations previously discussed. This position is based on a carefully defined sequence of events. After the withdrawal of LSM, the cessation of peripheral circulation, and therefore cerebral circulation, is observed leading to the loss of brain function. This state is maintained for five minutes before death is officially declared. In current French practice, however, the absence of circulation persists for approximately twenty minutes before A-NRP is initiated [3]. At the point NRP is initiated, the patient could not re-enter a trajectory of life. Before initiating NRP, targeted interventions are used to prevent or minimize any restoration of cerebral circulation, keeping any residual blood flow well below the thresholds that could allow for any recovery of brain function. The possibility of minimal restoration of the posterior cerebral circulation does not, in our view, undermine the determination of death. Under no circumstances could such marginal flow in the posterior cerebral circulation restore hemispheric function or consciousness. To suggest that this potential low-level cerebral reperfusion compromise the ethical validity of the dying process, the outcome of death as the best outcome for this patient, or the status of the donor as deceased appears to us ethically and clinically unfounded.

Building on the precedent analysis, the following section outlines a set of practical recommendations aims at ensuring that the use of NRP in cDCDD remains consistent with both ethical and clinical best practices [1]. All the technical strategies intended to prevent any restoration of cerebral circulation should be implemented prior to the NRP initiation. For A-NRP, this includes intra-aortic balloon occlusion; for TA-NRP, clamping the supra-aortic trunks and drainage of the cephalad ends into the thorax are required [2]. Throughout the NRP procedure, the absence of pressure in the left radial artery should be continuously monitored as an indicator of the effectiveness of the techniques implemented to exclude the cerebral circulation [3]. In parallel, to ensure that brainstem function loss remains permanent, specific clinical parameter should be observed, including the absence of pupillary reactivity - assessed either clinically or by pupillometry every 30 min - and the absence of diaphragmatic activity, at least until the administration of neuromuscular blockers used to facilitate organ procurement [4]. If objective signs of posterior cerebral function are detected during NRP–namely reactivity and/or diaphragmatic movements - corrective measures must be immediately undertaken to eliminate any restoration of cerebral circulation. This may include repositioning the intra-aortic balloon occlusion or checking the vascular clamps. Should these corrective measures fail, with persistence of signs of cerebral function–however partial, the organ procurement should be discontinued.

## Conclusion

The development of cDCDD - and more specifically the use of NRP- raises complex technical and ethical challenges. These issues deserve to be addressed with caution, as they have the potential to undermine the trust of stakeholders upon which deceased donation and transplantation systems fundamentally rely. In France, the perspective of heart procurement in cDCDD donors has prompted a renewed clinical and ethical reflection. Combining biomedical research and ethical deliberation, the French critical care community aims to ensure that this evolution in practice remains consistent with both scientific rigor, ethical clarity and end-of-life care.

## Data Availability

This work is a conceptual and ethical analysis; it does not include or rely on any original research data. Therefore, no data are available.
